# COVID-19 and Atherosclerosis: Is There an Association? Comment on Bielecka et al. From Systemic Inflammation to Vascular Remodeling: Investigating Carotid IMT in COVID-19 Survivors. *Viruses* 2025, *17*, 1196

**DOI:** 10.3390/v18020170

**Published:** 2026-01-28

**Authors:** Christian Saleh, Dorotea Gotler, Hrvoje Budinčević

**Affiliations:** 1 Independent Researcher, 4056 Basel, Switzerland; 2 Department of Neurology, National Memorial Hospital of Vukovar, 32000 Vukovar, Croatia; dvidakovic96@gmail.com; 3 Department of Neurology, Sveti Duh University Hospital, 10000 Zagreb, Croatia; hbudincevic@yahoo.com; 4 Department of Neurology and Neurosurgery, Faculty of Medicine Osijek, University Josip Juraj Strossmayer of Osijek, 31000 Osijek, Croatia; 5 Department of Psychiatry and Neurology, Faculty of Dental Medicine and Health, University Josip Juraj Strossmayer of Osijek, 31000 Osijek, Croatia

Bielecka et al. recently published a study titled “From Systemic Inflammation to Vascular Remodeling: Investigating Carotid IMT in COVID-19 Survivors” [1]. The study aimed to evaluate the potential impact of SARS-CoV-2 infection on the development of atherosclerosis (92 patients: 47 with COVID-19 and 45 controls) [1]. As a sonographic surrogate marker for preclinical atherosclerosis, the authors used the carotid intima-media thickness (CIMT), measured at baseline and 12–18 months apart [1]. Baseline CIMT values were comparable between the groups (0.85 mm vs. 0.78 mm), while the COVID-19 group exhibited a significantly greater increase in CIMT over time, with a median change of 0.13 mm compared to 0.05 mm in the controls (*p* = 0.018) [1]. The authors showed that elevated levels of C-reactive protein (CRP) and a higher triglyceride (Tg)-to-High-Density Lipoprotein Cholesterol (HDL) ratio were significantly associated with increased CIMT in the COVID-19 group [1]. Significant predictors of CIMT progression were age and heart rate in both groups [1]. The authors concluded that COVID-19 infection may accelerate the progression of subclinical atherosclerosis [1]. Several comments are needed to evaluate the results and conclusions of this study in a more balanced way. 1. Measurement location—CA section: The authors indicate that the CIMT was measured at the posterior (distal) CA wall in proximity to the carotid bifurcation [1]. This approach represents only a partial interrogation of the CA tree [1]. Because atherosclerosis develops in a focal and asymmetric manner, limiting measurements to a single predefined CA segment may fail to capture pathological arterial regions. Consequently, CIMT values obtained at a single CA location, or further in a predetermined location, may not accurately reflect the overall atherosclerotic burden of the vessel [2]. 2. Cardiac synchronization: Of paramount importance (international recommendations) is to synchronize the CIMT measure with the cardiac cycle, i.e., the end-diastolic phase [3]. CIMT measurements differ significantly between systole and diastole due to cyclic changes in arterial wall dimensions [4,5]. The authors did not report the use of cardiac synchronization [1]. The absence of cardiac synchronization may have introduced systematic measurement bias, as measurements may have been obtained in systole in some participants and in diastole in others, rendering measurements intra- as well inter-individually incomparable. 3. CIMT progression: When CIMT is measured at multiple time points, it is essential to specify how the baseline carotid artery segment was identified for follow-up because without consistent segment retrieval, observed differences may reflect normal, non-atherosclerotic wall variations rather than true atherosclerotic progression, leading to potentially misleading results [6]. 4. Intra-reader variability: Even if examinations were performed by a single examiner, the intra-reader variability needs to be mentioned, especially if, as in this case, measures were performed manually, as manual assessment may introduce variability and potential measurement bias compared with software-assisted measurements [6]. 5. IMT cut-off: The authors did not report which CIMT threshold, if any, they consider indicative of pathological thickening. Although no universally accepted cut-off exists, and the authors were focused on CIMT progression, various studies and guidelines propose different reference values depending on age, population characteristics, and measurement methodology [7,8,9,10,11,12]. 6. Blinding of the sonographer and/or analysis: The authors state that all measurements were performed by a single experienced sonographer, but it is not reported whether the sonographer was blinded to the participants’ COVID-19 status or to the time point (baseline vs. follow-up). Lack of blinding may have introduced measurement bias [13]. In conclusion: CIMT is a surrogate marker of preclinical atherosclerosis is a fast, non-invasive, and relatively inexpensive examination. However, it is a delicate measure, as its normal range is expressed within a submillimeter range. When using CIMT, examinations should be performed by highly trained operators, since the smallest inaccuracies (e.g., lack of cardiac synchronization, insufficient interrogation of the CA tree by asymmetric presentation of atherosclerosis) are sufficient to classify subjects into different CIMT categories, which, in turn, may affect clinical and pathophysiological interpretations. Therefore, a meticulous measurement protocol is essential, including a detailed description of the applied methodology.
